# Utility of the Right to Health for Addressing Skilled Health Worker Shortages in Low- and Middle-Income Countries

**DOI:** 10.34172/ijhpm.2022.6168

**Published:** 2022-02-07

**Authors:** Kenneth Yakubu, Seye Abimbola, Andrea Durbach, Christine Balane, David Peiris, Rohina Joshi

**Affiliations:** ^1^The George Institute for Global Health, Faculty of Medicine, University of New South Wales, Sydney, NSW, Australia.; ^2^School of Public Health, University of Sydney, Sydney, NSW, Australia.; ^3^Australian Human Rights Institute, Faculty of Law, University of New South Wales, Sydney, NSW, Australia.; ^4^Discipline of Paediatrics, School of Women’s and Children’s Health, University of New South Wales, Sydney, NSW, Australia.; ^5^School of Population Health, Faculty of Medicine, University of New South Wales, Sydney, NSW, Australia.

**Keywords:** Health Workforce, Right to Health, Global Health

## Abstract

**Background:** As a fundamental human right, the right to health (RTH) can influence state actors’ behaviour towards health inequities. Human rights advocates have invoked the RTH in a collective demand for improved access to essential medicines in low- and middle-income countries (LMICs). Similarly, scholars have used the RTH as a framework for analysing health problems. However, its utility for addressing skilled health worker (SHW) shortages in LMICs has been understudied. Realising that SHW shortages occur due to existing push-and-pull factors within and between LMICs and high-income countries (HICs), we sought to answer the question: "how, why, and under what circumstance does the RTH offer utility for addressing SHW shortages in LMICs?"

**Methods:** We conducted a realist synthesis of evidence identified through a systematic search of peer-reviewed articles in Embase, Global Health, Medline (Ovid), ProQuest – Health & Medical databases, Scopus (Elsevier), Web of Science (Clarivate), CINAHL (EBSCO), APAIS-Health, Health Systems Evidence and PDQ-EVIDENCE; as well as grey literature from Google Scholar.

**Results:** We found that the RTH offers utility for addressing SHW shortages in LMICs through HIC state actors’ concerns for their countries’ reputational risk, recognition of their obligation to support health workforce strengthening in LMICs, and concerns for the cost implication. State actors in LMICs will respond to adopt programs inspired by the RTH when they are convinced that it offers tangible national benefits and are not overly burdened with ensuring its success. The socio-economic and institutional factors that constrain state actors’ response include financial cost and sustainability of rights’-based options.

**Conclusion:** State and non-state actors can use the RTH as a resource for promoting collective action towards addressing SHW shortages in LMICs. It can also inform negotiations between state actors in LMICs and their HIC counterparts.

## Background

The right to health (RTH) is a fundamental human right. It captures the entitlement of everyone to enjoy the highest attainable standard of physical and mental health. Access to health services, and skilled health workers (SHWs) are essential aspects of realising this right.^1,2^ The United Nations Committee on Economic, Social and Cultural Rights has described core obligations that governments should progressively fulfil to respect, protect and fulfil this right.^1^ These core obligations include (1) ensuring access to health facilities, goods and services in a non-discriminatory manner; (2) access to essential medicines; (3) developing and implementing an evidence-based national public health plan that addresses the health needs of its entire population through a transparent and participatory process; (4) the use of RTH indicators in monitoring this strategy; and (5) promoting conditions that improve the social determinants of health (including essential food, shelter, housing, sanitation and potable water).^1,3^ Backman et al described a 72-indicator framework for assessing the RTH features of health systems^4^ including four that directly impact maintaining and retaining an optimal skilled health workforce. These include adequate remuneration, sustainable national financing for an essential public health system, international assistance and cooperation, and measures to prevent violation of populations’ RTH.

During the HIV pandemic, human rights advocates mounted pressure on governments worldwide to see the effects of the disease in low- and middle-income countries (LMICs) as a violation of human rights. Seeing that populations in LMICs had limited access to necessary health services, the RTH was used to give normative force to collective demands for improved access to essential medicines.^5,6^

At the time, the United States, as a global world power, came under much criticism because its foreign policy did little to address the HIV pandemic in LMICs. Its decision to support measures to improve access to HIV treatment in LMICs was influenced by a combination of international solidarity, social pressure, and concerns for a public health threat from an uncontrolled HIV pandemic in LMICs.^5^ Seeing the role that invoking human rights played in mobilising international solidarity to control the HIV pandemic, experts have applied its framework in studying various health problems.^7^ Though access to health workers has come up indirectly within efforts to address the HIV pandemic; there is a knowledge gap on the potential of using the RTH to address SHW shortages in LMICs.

Efforts of LMIC governments to address health workforce deficits in their countries include focusing on improving entry into the workforce, eg, training schemes aimed at increasing the number of appropriate people entering the health workforce. To improve and maintain the current stock of SHWs, LMIC governments have focused on compulsory service requirements, regulating the scope of practice, distribution of personnel, financial incentives, and personal and professional development.^8^ For retention of SHWs, state actors in LMICs have favoured restrictive/regulatory mechanisms and incentives.^9,10^ High-income country (HIC) have provided technical and financial aid to support the health systems of LMICs, and enacted measures to end active recruitment of LMIC-trained SHWs.^11^

However, compared to policies focused on entry into the workforce and maintenance of the current stock of health workers, controlling the exit of SHWs has proved far challenging to achieve due to HIC’s reliance on foreign-trained SHWs, and various push factors (including insecurity, poor remuneration, poor work conditions, and poor access to safe/potable water) in LMICs.^12-14^ This study aimed to generate an understanding of how the RTH influences the response of state actors towards addressing SHW shortages in LMICs and describe the social systems that may enable or constrain its use and effects. Using a realist review, we sought to answer the question: how, why, and under what circumstance does the RTH (ie, its implicit or explicit presence at the initiation and implementation of policy initiatives) offer utility for addressing SHW shortages in LMICs?

## Methods

###  Review Approach

We considered the realist approach for evidence synthesis best suited to the review question and submitted a protocol to the PROSPERO registry (ID number CRD42019139372). A realist review produces program theories (propositional statements or causal connections) that explain what it is about interventions/programs that work, under what conditions, and why.^15^ These theories or “causal connections” are often expressed using a C-M-O configuration. “C”refers to the context (characteristics of the individuals of interest and their surrounding circumstances), “M” refers to mechanisms (how these individuals of interest through their reasoning and behaviour, respond to the resources provided by the intervention), and “O” refers to outcome patterns.^16^ Rather than focus only on intervention types and their outcomes, a realist methodology also argues for the agency of individuals. It links the success/failure of programs/interventions to their reasoning and behaviour (ie, mechanisms).^15,17^ We established a priori that the RTH is a conceptual resource that operates through actors’ reasoning and behaviour. We therefore aimed to design, test, and refine an initial program theory for how its presence or lack thereof yields workforce initiatives/policies aimed at addressing SHW shortages in LMICs. We relied on the World Health Organization (WHO) work lifespan for a definition of desired outcomes – change in (*i*) enrolments into undergraduate or graduate health training posts (O_1_), (*ii*) graduation rates (O_2_), (*iii*) recruitments into the health workforce (O_3_), (*iv*) total stock of SHWs in a country (O_4_), and (*v*) retention/migration rates (O_5_)^18^, and focused not on the workforce intervention types, but on explaining semi-predictable (demi-regularities) patterns of reasoning and behaviour among state-actors in LMICs and HICs.^19,20^

###  Search Strategy for the Initial Program Theory

We conducted an initial non-systematic literature search in Google Scholar and PubMed and held discussions among the authorship team (and their networks, especially professionals actively involved in healthcare delivery in LMICs) to understand how the RTH may offer utility for addressing SHW shortages. The findings from our search and discussions informed the development of an initial program theory (see Table 1), a singular context-mechanism-outcome (CMO) which was subsequently tested/refined and expanded following a systematic search of the literature.

**Table 1:** Initial Program Theory for How the Right to Health Impacts Skilled Health Worker Shortages in Low- and Middle-Income Countries

**Context **	**Mechanism**	**Outcome**
In LMICs with SHW shortage due to a lack of resources, poor allocation of resources for public health services (including SHW training); internal political conflicts; poor working conditions and migration of SHWs to HICs… AND/OR In HICs that contribute to LMIC SHW shortages through aggressive recruitment and incentivisation; minimal or no international assistance for LMICs’ health service needs… When people are aware of the RTH and express their dissatisfaction to a responsive government about public health challenges resulting from SHW shortages in LMICs (eg, long waiting times at hospitals and preventable high mortality rates)…	…then, the mounting internal and/or external pressure (either through legal action, protests, withdrawal of political support, or advocacy) or inherent dissatisfaction with the status quo among state actors, will trigger behaviour aimed at guaranteeing the RTH of people in LMICs. LMIC governments will increase the allocation of resources for training and/or recruiting more SHWs, and choose to maintain/enhance the level of health and social services in their countries by seeking external assistance. AND/OR HICs governments will support measures aimed at strengthening the health systems of LMICs and refrain from activities that violate their RTH.	…therefore, the system of accountability that results from invoking the RTH will lead to an increase in the production of SHWs, a reduction in the rate of migration and an increase in the total stock of SHWs in LMICs.

Abbreviations: LMIC, low- and middle-income country; RTH, right to health; HICs, high-income countries; SHW, skilled health worker.

###  For the Refined Program Theory

To derive a refined program theory, we used the terms #1[“Emigration and Immigration”] AND #2[Health Personnel OR Health workers] AND #3[“Recruit” OR “Retention” OR “Regulation”] AND #3[“Developing countries” OR “low- and middle-income countries” OR “LMICs”] and conducted a systematic search of peer-reviewed articles in Embase (Ovid), Medline (Ovid), Global Health (Ovid), ProQuest – Health & Medical databases, Scopus (Elsevier), Web of Science (Clarivate), Embase (Ovid), CINAHL (EBSCO), APAIS-Health: Australian Public Affairs Information Service – Health, Health Systems Evidence and PDQ-EVIDENCE. For grey literature, we included the first 300 documents from Google Scholar. While searching the peer-reviewed literature, we set the date range from the inception of each of the databases to March 2021. We have provided details of the search strategy in Supplementary file 1.

###  Inclusion Criteria

 To be included, studies had to report, at minimum, a policy (either already implemented or modelled) aimed at addressing SHW migration, their recruitment and retention in a low-income or middle-income country. We defined SHWs as doctors, nurses, midwives, and pharmacists irrespective of their specialisation.

###  Exclusion Criteria

 We excluded any article that did not mention a specific policy or focused only on describing the problem of SHW shortages.

###  Quality Appraisal

To assess the quality of each primary study in a realist synthesis, it is essential to consider their relevance and rigour.^21^ Based on the description of relevance by Pawson, we considered whether each primary study provided helpful information for testing or refining the initial program theory.^21^ To establish the rigour of each study, we considered whether the information they provided was plausible. We did this by examining the extent to which the methods and findings align, and whether the authors’ inferences were justifiable based on similar studies, or what we know about the subject matter.^21^ To reduce bias, two authors (KY and CB) reviewed each article independently, discussed disagreements, and reached a consensus for all articles.

###  Data Extraction, Categorisation, and Appraisal

The authors (KY and CB) independently screened the abstracts of articles identified from the search strategy and the full text of the articles that met the eligibility criteria. The two review authors discussed and resolved any disagreement regarding the eligibility of an article at each stage of the review process. After that, we included 68 articles in the review (Figure). We piloted the data extraction process by reviewing three articles together. After standardising the review and data extraction process, each author independently extracted data for the remaining articles. To determine whether the RTH preceded an intervention/policy and characterised its implementation, we mapped the information provided in the articles to a list of RTH items. We derived this list by combining core obligations of State parties as specified by the General Comment 14,^3^ with four RTH indicators adopted from Backman’s framework.^4^ The four RTH indicators include adequate remuneration, sustainable national financing for an essential public health system, international assistance/cooperation, and measures to prevent violation of a population’s RTH. See Box 1 for a complete list of RTH items.

**Figure:**
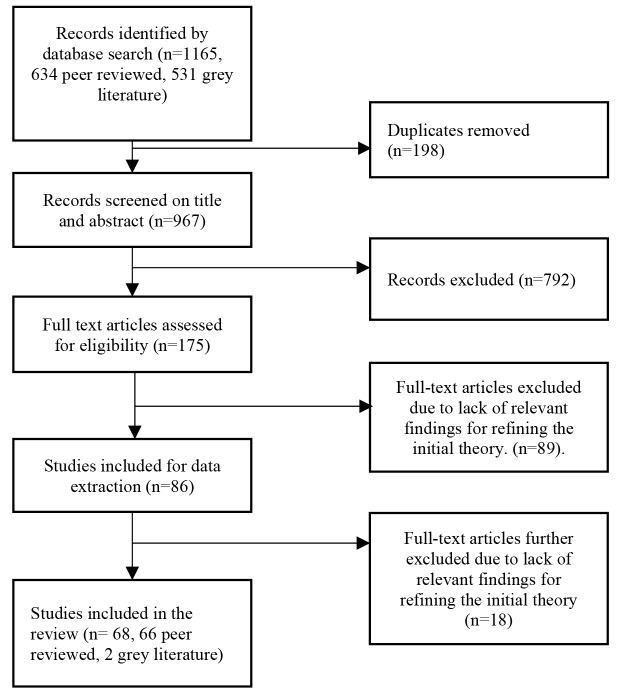
Document Flow Diagram.

**Box 1. Right to Health Items**

1. Does it mention access to good quality health facilities, products, and services in a non-discriminatory manner, especially for vulnerable or marginalised groups?

2. Does it mention access to the minimum essential food nutritionally adequate and safe to ensure freedom from hunger to everyone?

3. Does it mention access to basic shelter, housing, and sanitation, and an adequate safe and potable water supply?

4. Does it mention the provision of essential drugs from time to time, as defined under the WHO Action Programme on Essential Drugs?

5. Does it mention an equitable distribution of all health facilities, goods, and services?

6. Does it mention the adoption of a national public health strategy and plan of action based on epidemiological evidence, addressing the health concerns of the whole population?

7. As per (6) above, is there a plan to use the RTH indicators in periodic monitoring of this public health strategy; based on transparency, accountability, and participation of the population (including the marginalised)?

8. Does it mention a national health workforce strategy (including a plan for self-sufficiency of skilled health personnel)?

9. Does it mention adequate remuneration of skilled health personnel (including incentives for working in rural areas)?

10. Does it mention sustainable national financing for an essential public health system?

11. Is there mention of international assistance and cooperation (eg, economic or technical) to promote and protect population health and strengthen health systems?

12. Are there measures to prevent violations of population health by state and non-state actors, such as private persons (including recruiters of health workers), employers and enterprises?

Abbreviations: RTH, right to health; WHO, World Health Organization.

###  Theoretical Framing

To arrive at underpinning explanatory mechanisms of an outcome, Danermark et al described six steps ranging from (*i*) a concrete description of the event, (*ii*) analytical resolution into its sub-components, (*iii*) abduction or theoretical redescription of what may have led to the event, (*iv*) retroduction, (*v*) comparison between different theories and abstractions, (*vi*) concretisation and contextualisation.^22^ For this review, we adapted these six steps and were informed by Abimbola et al^23^ andJagosh.^24^ Our adaptation merged Danermark’s steps (iv-vi) and was easier to apply in this study. We have provided a stepwise account of the realist analysis in Box 2. Describing this briefly, KY and CB documented the interventions and their outcomes. We then searched each of the articles for descriptions of what led to the adoption of workforce initiatives/policies (including the role of RTH) and annotated components that were consistent with items in the RTH framework. We then explored reasons why the implementation of an initiative improved or failed to improve SHW shortages and whether these were related to the relevant components of the RTH framework.

**Box 2. Steps for the Realist Analysis**

**Step 1: Description** – KY and CB read each article to gain familiarity with the studies and identify relevant outcomes. Using a standard data extraction form on an excel spreadsheet, we copied and pasted verbatim, sections of the articles that reported the outcomes of interest. The outcomes include any information on a change in the number of enrollments into undergraduate or postgraduate medical training, graduation, recruitment into the workforce, the current stock of the health workforce, and retention/emigration rates that occurred after implementation of the workforce policy/program.

**Step 2: Resolution** – KY and CB also identified and extracted verbatim, relevant information on the context (ie, enablers and constraints of the desired outcomes) documented in each article. We considered a piece of information as context if it described peculiar characteristics, socio-economic circumstances, formal or informal rules peculiar to individuals, groups, institutions, and countries that influenced actions, decisions, and relationships among state actors.

**Step 3: Abduction** – For each context-outcome pair, KY studied state actors’ documented decisions and actions, including what led them to consider a policy/workforce option (ie, their reasoning) and the types of policies implemented (ie, their behaviour). These documented decisions and actions were then compared with the list of RTH items (Box 1) and reconstructed into plausible patterns or rules for reasoning and behaviour. KY considered and identified keywords from the context-outcome pairs. He combined these keywords with the terms Theor* OR Framework OR Models and conducted a search in Google Scholar. From the first 300 results, KY found 53 relevant to the empirical data. He reduced these to 4 articles that offered three theoretical perspectives that provided the best-fit explanations for the context and outcomes (see Supplementary file 4). These explanations were discussed with SA, CB, DP, RJ, and AD.

**Step 4: Retroduction** – Armed with a list of explanations, KY re-examined the patterns or rules of reasoning and behaviour constructed in Step 3, examined each contextual enabler and constraint, moving back and forth till he achieved a consistent explanation for state actors’ reasoning and behaviour for each group of observed outcomes and context pairs. All the other co-authors then reviewed these explanations.

## Results

 Our initial search yielded a total of 1165 articles. After removing duplicates, we reduced this to 967. We have shown the overall study selection process in Figure.

 A total of 68 publications met the inclusion criteria after full-text review. These represented health workforce policies/programs that involved Angola, Botswana, Burundi, Cameroun, Eswatini, Ethiopia, Ghana, Kenya, Lesotho, Libya, Malawi, Mali, Mauritius, Mozambique, Nigeria, South Africa, Sudan, Tanzania, Uganda, Zambia, and Zimbabwe (Africa); Iraq, Saudi Arabia (East Mediterranean region); the Caribbean region; Brazil, Cuba (South America); Afghanistan, Australia, Bangladesh, Cambodia, Fiji, India, Indonesia, Kazakhstan, Laos, Nepal, Papua New Guinea, Philippines, Thailand, and Vietnam (Asia-Pacific); Canada and the United States (North America); France, Germany, Norway, the Netherlands, Sweden, and the United Kingdom (Europe).

The RAMESES publication standards for realist synthesis guided the reporting of our findings (see Supplementary file 2).^25^ In describing our findings, we mentioned the workforce outcomes and where the workforce initiatives were implemented (whether solely by HIC or LMICs). We also mentioned the associated context (socio-economic, institutional, or individual factors that constrained the outcomes) and the mechanisms (ie, the reasoning and behaviour of relevant actors and how it aligned with a RTH item – denoted by a number).

We did not focus on the mechanisms triggered by the workforce intervention types (ie, at the level of the SHW) but on the program theories that explain how state actors respond to an invocation of the RTH. We have provided a summary of the program theories using CMO configurations in Table 2. We have also provided details of the articles and the corresponding RTH items in Supplementary file 3.

**Table 2:** Program Theories on How the Right to Health Offers Utility for Addressing Skilled Health Worker Shortages in LMICs, Considering State Actors in HICs and LMICs

	**Context **	**Mechanism**	**Outcome**
**HICs**			
CMO1	HICs have an unmet demand for SHWs and contribute to SHW shortages in LMICs through aggressive recruitment/incentivisation; no/minimal international assistance per LMIC's health service needs. If HICs have a centralised workforce management system to communicate ethical guidelines and can seek alternative sources (other than countries with critical shortages) from which to recruit SHWs, then when there is a sustained demand for the RTH from within and outside its borders, particularly by LMIC governments with globally revered state actors,	they will respond due to concerns for reputational risk, and the need to restore social approval.	HIC governments will then avoid unethical recruitment of SHWs from LMCs, which will reduce the number of SHW migration from LMICs.
CMO2	If there is a continuing expectation from within and outside HICs about their role in promoting the RTH in LMICs, and if meeting this expectation incurs high financial cost and is unsustainable,	then even though HIC governments recognise their obligation to LMICs, they will either attempt to provide time-bound support that is limited in scope or avoid any commitment altogether.	Such HIC efforts may have no impact on SHW shortages in LMICs or, due to the limited support, may lead to an initial increase in the number of enrollments into health training programs in LMICs, recruitments, and their retention in the country, which subsequently declines.
**LMICs**			
CMO3	Many LMICs face SHW shortages due to poor allocation of resources to provide public health services (including SHW training), internal political conflicts, poor working conditions, and migration of SHWs to HICs. If there is an existing collaboration among relevant stakeholders within and outside LMICs, and each of them show commitment to policies/programs of mutual benefit,	then when LMIC governments are presented with workforce initiatives inspired by the RTH, they will be willing to support such because they feel less burdened with ensuring its success, and with negotiating competing interests among existing stakeholders. LMIC governments will then favour self-regulation among stakeholders concerned with these initiatives and seek to complement their efforts to strengthen the skilled health workforce and improve healthcare access.	Self-regulation among relevant stakeholders and a corresponding action by an LMIC government will lead to improved work conditions for SHWs, increased enrollment in health training programs, and those willing to join the health workforce. The number of SHWs motivated to stay and offer health services in LMICs will increase, thus increasing their retention.

Abbreviations: LMIC, low- and middle-income country; RTH, right to health; HICs, high-income countries; SHW, skilled health worker.

###  Program Theories From High-Income Countries 

####  HIC State Actors’ Concerns About Reputational Risk

**CMO1**. If there is a sustained expression of discontent by the international community about the impact of foreign recruitment on access to healthcare services in LMICs, then state actors in HICs will be concerned about the reputational risk of their countries (M) and will take actions leading to a reduction in SHW migration (O_5_). This action by state actors in HICs is more likely to occur with the involvement of civil society in both LMICs and HICs, and individuals in LMICs who have achieved global recognition for the values they stand for. A change in HIC recruitment behaviour is further enabled when the workforce is centrally managed at the national level (C_1_) and when the government has access to alternative sources from which to recruit SHWs (C_2_).

The international community has repeatedly expressed concerns about the negative impact of HIC recruitment practices on access to healthcare services in LMICs (RTH 12). Compared to LMICs that had little negotiating power and posed little reputational risk,^26,27^ HIC governments came under significant pressure to change their recruitment policies (RTH 12) when interactions with LMICs featured civil society organisations in both LMICs and HICs, and elder statesmen that were globally revered.^28-34^ HIC state actors signalled a change in recruiting behaviour by implementing visa restrictions, an ethical code of conduct to guide recruitment practices, and reduced issuance of work permits to LMIC-trained SHWs.^34-36^ This action by HIC state actors eventually led to a reduction in SHW migration from LMICs (RTH 12).

A change in recruitment behaviour was enabled when HIC governments operated a centralised health workforce management system (eg, a single national system for the recruitment of foreign SHWs) such that it was possible to implement an ethical code of conduct.^34,37,38^ It was also easier for HICs to reduce recruitment of SHWs from LMICs when a global financial crisis discouraged government spending,^39,40^ domestic production of health professionals in HICs increased,^38,41^ recruitment of SHWs from other sources apart from those with critical shortages was possible,^37,41^ or when other political interests and language requirements made recruitment from LMICs less desirable.^41^

In the absence of alternative options for meeting the rising demands for health services in their countries, HIC government’s concerns for reputational risk waned, and recruitment from LMICs continued.^30-33,35,36,42-46^

####  Obligation and Cost-Concerns for Supporting Health Workforce Strengthening in LMICs 

**CMO2. **When relevant stakeholders in LMICs and HICs demand improved access to healthcare services in LMICs, then HIC governments may recognise their obligation to achieve this through health workforce strengthening (M_1_). Such recognition is easier for HICs where a shared understanding of this obligation has been reinforced over time through engagements between relevant actors within and outside their borders (C_1_). However, if the financial cost of providing this support is high and unsustainable (C_2_), then they will either avoid commitment to workforce strengthening in LMICs (M_2_) or provide support that is time-bound and limited in scope (M_3_). This support by HIC governments may have no impact on SHW shortages in LMICs or lead to an initial (but unsustainable) increase in the number of enrolments into health training programs (O_1_), recruitments (O_3_), and their retention in the country (O_5_).

Governments of LMICs, health professional groups, and the news media in both HICs and LMICs have repeatedly argued that HIC governments should do more to protect access to health services in LMICs through health systems strengthening (RTH 11).^34,38,39,47-52^ For HICs with a history of recognising an obligation to LMICs, existing expectations from their citizens and the international community has enabled continuity of such supportive behaviour. As a result, HIC governments have provided funds for building more training institutions in LMICs (RTH 8, 11); scholarships for enrolment into speciality training programs that are a priority to populations in LMICs (RTH 6, 8, 9)^47,48,53-58^; supported recruitment, remuneration and the provision of incentives for SHWs (RTH 8, 9,11). These efforts have led to increased enrolment into professional health training programs, expansion of the existing health workforce, and retention of SHWs in LMICs.^35,44-46,49,51,53-55,59-64^

These outcomes were further enabled in LMICs with a centralised workforce management system, acting as the single point of entry and diffusion of HIC technical and financial support.^49,51^ It was also easy for HIC governments to commit to improving access to health services in LMICs when they identified mutually beneficial arrangements with LMIC governments (eg, time-limited placements in a HIC, health worker training arrangements to meet workforce needs in a HIC and LMIC).^56,65^ Collaborating with HICs often imposed an additional burden on LMIC health staff, who had to add the management of those partnerships to their routine health services.^47,49,51,58^

For LMIC governments that relied mainly on HIC support for their infrastructural development, it was difficult to sustain these health workforce outcomes (ie, increased enrollment, expansion of the existing health workforce, and retention of SHWs in LMICs).^45,53,62,66,67^ For many LMICs, local training capacity for health workers was limited and required trainees to travel abroad. When present, training programs often led to an overproduction of health workers who either remained in the urban areas or migrated abroad. In other instances, available training staff were overstretched, raising concerns about the quality of training offered.^48-51^ Yet in other LMICs, high rates of HIV among young SHWs constrained efforts to increase the stock of SHWs, resulting in a predominantly ageing workforce.^68-70^ Many LMICs also experienced an economic downturn and had a cap on public employment, such that external funding constituted a significant part of health expenditure.^38,49,51^ As a result, it was common for HIC support to be time-bound, focused on vertical programs for diseases that were perceived to be public health threats (eg, HIV/AIDS), where outputs linked to inputs could be demonstrated and evaluated.^51,60^

###  Program Theories From Low- and Middle-Income Countries

####  The Burden of Negotiating Rights-Based Workforce Interventions

**CMO3: **If LMIC state actors are presented with policy options inspired by the RTH and are not overly burdened with negotiating the trade-offs required to ensure its success (M_1_), then they will be motivated to work with these options. This response by LMIC state actors is possible when SHWs lead multi-stakeholder engagements to reduce the impact of migration on access to healthcare services (C_1_). Implementation of these policy options leading to a reduction in the rate of SHW migration (O_5_), increased enrolment into health professional training programs (O_1_), and an expansion of the current stock of SHWs in LMICs (O_4_) is easier to achieve when there are existing collaborations between state and non-state actors within and outside their countries (C_2_), and because these stakeholders are willing to share in the financial cost of achieving a compromise between SHWs’ right to migrate, and citizens’ access to health services (C_3_).

Following public concerns about availability, safety, and quality of health services in LMICs, SHWs remained committed to promoting their rights to migrate and took a leadership role in promoting initiatives to reduce the impact of SHW migration on access to health services (RTH 12).^56,65^ They played a gate-keeping role for colleagues who sought verification for international employment, which reduced the rate of SHW migration to a HIC.^71^ Leveraging on existing regional and international networks, they designed initiatives to increase enrollment into training programs for health workers, their retention in LMICs, while ensuring that their countries benefit from SHW migration (RTH 8,11,12).^56,65^ Since SHWs provided leadership for communicating the benefits of these rights-based initiatives and were willing to bear the cost of negotiating a compromise between relevant stakeholders, it was easy for state actors to support their recommendations and accept them as part of their programs for achieving health workforce sustainability.^56,65,71^ Where interactions between multiple stakeholders in LMICs was absent, each pursued its interests, thus weakening existing capacity for monitoring and implementing health workforce initiatives.^26,27,44,45,54,55,59-61,64,66,67,70,72-78^

Such interactions between state and non-state actors in LMICs were further constrained in the presence of political instability. During such periods, migration spikes were observed, leading to larger social networks abroad and a constant pull-effect on remaining SHWs.^66,72,74^ Even when there was a synergy between state and non-state actors, but the predominant reasoning was to make economic gains from the migration of SHWs to HICs, adopting a holistic approach to SHW shortages was difficult.^70,76,79-81^ Hence, where there was political stability and state actors were embedded in a network of stakeholders with a rich uptake of the RTH and a willingness to negotiate competing interests around increasing access to health services; it was easier to achieve increased retention of SHWs.^26,56,65,79,82^

####  State Actor’s Aversion for Disruption in Healthcare Services

**CMO4: **Since disruption in the provision of health services will reflect poorly on state actors in LMICs (M_1_), they are willing to avoid or resolve industrial strike actions in the health system by taking steps that improve work conditions, favour an expansion of the existing workforce (O_4_), increase enrolments into health training programs (O_1_) and improve retention (O_5_). This response by LMIC state actors is made easier with support from other LMICs and constrained in the presence of poor socio-economic conditions (C_1_).

When SHWs in LMICs strike to protest worsening working conditions, they draw state actors’ attention to a worsening health workforce crisis. Even though the agitations were not primarily concerned with improving access to health services, LMIC governments still sought to resolve the prevailing labour crisis. They responded by increasing remunerations for health workers, providing incentives, improving work conditions and increasing training programs.^60,61,63,83^ This was easier to achieve in LMICs with health professional groups mobilised towards advocating for improved working conditions.^71,83^ It was constrained by worsening social and health inequities, insecurity, and national economic difficulties that exacerbated existing trends for SHW migration.^68,69^

####  Instances Where No Intervention/Trigger Factor Was Found

In other instances, external triggers did not precede state actors’ response to worsening SHW shortages in LMICs. This finding may especially be true in countries where commitment to public service and an egalitarian health system are encouraged.^70,84^ Without evidence of an implicit or explicit invocation of the RTH or strike action by SHWs in LMICs, some LMIC state actors have shown concerns for improving access to health services in their countries and addressing existing health inequities that arise due to shortages of SHWs (RTH 8).^44,50,54,66,70,71,74,75,77,79,84-91^

Without external triggers, LMIC state actors have considered options to expand health professional training systems,^59,70,72,79,90,92,93^ and improve service delivery from an existing stock of SHWs (eg, performance-based payments and a locum scheme).^63,90^ They have also implemented policies to expand their stock of SHWs by increasing remunerations and offering work incentives, extending health workers’ retirement age, and sending SHWs to provide health services in rural areas. In addition, they have offered continuous professional development to existing SHWs and recruited from LMICs with health worker surplus (RTH 5,8,9).^26,27,60,62,64,67-70,72,73,78,85-87,89-91^ Where such LMICs were unable to fund their own training program, they leveraged on existing regional collaboration to support and achieve training initiatives (RTH 8,11).^41,53,56,62,67,70,72,74,75,79-81,88^

## Discussion

We found that the RTH influenced the response of HIC state actors to SHW shortages in LMICs through two mechanisms. These include (*i*) concerns about their reputational risk when their recruitment practices were criticised for impacting access to health services in LMICs, and (*ii*) a combination of recognising their obligation to support health workforce strengthening in LMICs, and concerns for the cost implication.

In LMICs, we found that programs inspired by the RTH elicited a favourable response from state actors if it offered a tangible national benefit and if they were not overly burdened with the responsibility for ensuring its success. We also found that in the absence of the RTH as a conceptual resource, state actors in LMICs still responded to SHW shortages because they wished to avoid/address labour crises in the health system or were influenced by an existing commitment to public service in their countries. Each of the mechanisms inspired by the RTH was enabled or constrained by a broad range of contextual factors summarised in Table 2.

A common view of rights-based approaches for improving access to health services is its expression through protests or a judicial process.^94-96^ In our review, we did not find any judicial process through which the RTH offered utility for addressing SHW shortages in LMICs. Instead, we found that interactions about the RTH followed a social process involving state and non-state actors. Where state actors in HICs were exposed to enough social pressure, it led to a necessary response for addressing existing SHW shortages in LMICs.^28,34^

Such responses by HIC state actors’ relied on the ability of claimants to expose them to reputational risk. HIC state actors’ concern for reputational risk relied on recognising their obligation to the international community and concerns for their countries’ international social standing. However, the utility of the RTH may be constrained when in the face of limited financial resources, state actors in HICs have to prioritise meeting the workforce needs of their country (the smaller community they belong to) over those of other countries (the international community).^97^

Where state actors showed a positive response to SHW shortages in LMICs, we also found that the RTH was not always the motivation.^66,74,75,81,84^ This supports other studies where prevailing norms of social justice and reciprocity offered utility for addressing health inequities in the absence of rights-based interventions.^97-99^ Even though the concepts of community, social justice, and human rights are often understood as separate entities, our findings reiterate those from other studies that have demonstrated how a sense of community and social justice can have a synergistic effect on rights-based approaches.^97,98,100-102^

###  Strengths and Limitations 

To our knowledge, this is the first realist review that examined the utility of the RTH for addressing SHW shortages in LMICs. We included studies that implicitly referred to the RTH. This decision to include articles with implicit reference to the RTH is consistent with methods used by other authors where they mapped portions of government interventions to the normative components of the RTH.^96,103,104^ The UN High Commission on Human Rights has described the use of a subjective approach (ie, perceptions, opinion and judgement) for examining state actors’ reasoning and behaviour towards the RTH as valid, provided there is transparency in the approach.^105^

 The RTH items we observed in this review were limited to the health system and included access to an equitable distribution of health facilities, goods, and services (RTH 1,5). It also includes access to essential medicines (RTH 4), a national public health plan based on epidemiological evidence (RTH 6), a workforce strategy, adequate remuneration/incentives (RTH 8,9), international cooperation and assistance (RTH 11), and steps taken to prevent violation of a country’s public health (RTH 12). We may have missed broader efforts (eg, economic, social, and political) that address factors impacting SHW migration.

Workforce interventions are dynamic and emergent, and a complete understanding of their content may have been limited by the brief descriptions we provided in each paper. However, our study is consistent with the realist methodology, which seeks to explain analytically derived mechanisms rather than the actual contents of interventions.^106^ The geographical spread our review represents suggests the reach of our program theories, where it can be tested and further refined.

Our decision to focus on the behaviour of state actors (instead of SHWs) aligns with the United Nations Committee on Economic, Social and Cultural Rights. The United Nations committee places the obligation for the realisation of the RTH on state actors and their collaboration with non-state or foreign actors who can provide support.^1,3^

Seeing that a similar study examined interventions aimed at addressing SHW shortages by focusing on their distribution within countries, our study contributes to the literature by addressing this from a perspective of international migration.^107^ While our study acknowledges SHW shortage as a global problem, to promote health equity, we have focused on LMICs where a critical shortage of SHWs exist.

###  Implications for Programs and Policy-Makers

Judicial systems in many LMICs do not yet offer robust mechanisms for protecting the RTH.^96,108^ Though improving the justiciability of the RTH may improve its utility for addressing SHW shortages in LMICs, our findings suggest that a combination of legal and non-legal mechanisms may be required for promoting accountability in the global governance of the health workforce and health professional migration. Promoting accountability among state and non-state actors worldwide is even more pertinent as the coronavirus disease 2019 (COVID-19) pandemic increases concerns about achieving a sustainable global health workforce.^109^

 Furthermore, state and non-state actors in LMICs may need to rely on the RTH as a tool for increasing reputational risk for HICs and negotiating mutually beneficial workforce arrangements. However, the results of such negotiations may be short-lived in LMICs that lack strong public institutions and democratic systems for promoting accountability among relevant stakeholders.

Since human rights are interdependent and indivisible, the RTH by itself will offer little utility for addressing SHW shortages in LMICs and will require the presence of other socio-economic (for example, right to education, adequate food, clothing, and housing) and political rights.^110^ Hence, appropriate use of the RTH for addressing SHW shortages in LMICs will require capacity building for strengthening health, social, economic, and political systems in LMICs. Such capacity-building efforts will also need to consider SHWs not just as victims of push and pull migration forces but also as potential leaders in engagements between relevant stakeholders.

## Conclusion

 As a conceptual resource, the RTH (either explicitly or implicitly) can influence the reasoning and behaviour of state actors towards addressing SHW shortages in LMICs. Its utility for addressing these shortages may be realised when state actors in HICs are exposed to reputational risk. Getting the desired response from HIC state actors will require a high capacity for global health diplomacy among relevant stakeholders in LMICs, and their ability to lean on the RTH as a resource for negotiating mutually beneficial workforce initiatives. Realising the utility of the RTH for addressing SHW shortages in LMICs will also require multi-stakeholder engagements.

 Such engagements should include SHWs not just as victims of the push and pull forces of migration but as relevant stakeholders willing to use the RTH as a resource for promoting collective action within and between LMICs and HICs. It will also require the willingness of LMIC state actors to harness available resources and expertise within and outside their countries for strengthening health, social, economic, and political systems.

 Simultaneous considerations for reducing the transactional cost of maintaining these engagements, incorporating the RTH into domestic judicial systems, and implementing the policy options they yield, will be needed. It will also require a critical number of state and non-state actors in LMICs and HICs who are committed to the concepts of social justice and an inclusive global community.

## Acknowledgements

 We would like to thank Drs. Emmanuel Laabes (Australia), Joshua Sule (Nigeria), Klaus von Pressentin (South Africa), Innocent Besigye (Uganda), and Murtaza Haiderbhai (Tanzania) for their feedback on the initial program theory for this review.

## Ethical issues

 Since this is a review of the existing literature, no ethical approval was required.

## Competing interests

 Authors declare that they have no competing interests.

## Authors’ contributions

 KY and CB conducted the literature search, screening, and extraction of relevant data. KY conducted the realist analysis and received feedback from all the co-authors. KY wrote the first draft of the manuscript, which all co-authors reviewed and made contributions for improving its intellectual content. All authors agreed on the final draft. RJ is the senior author for this study.

## Disclaimer

 The authors alone are responsible for the views expressed in this publication and these do not necessarily represent the views, decisions, or policies of The George Institute for Global Health, University of New South Wales (UNSW), Australian National Heart Foundation, or the Australian National Health and Medical Research Council.

## Funding

 KY is supported by the Scientia PhD Scholarship at UNSW. RJ is a Scientia Fellow at UNSW and is supported by a Future Leadership Fellowship from the Australian National Heart Foundation (102059). DP is supported by fellowships from the National Health and Medical Research Council of Australia (1143904) and the Heart Foundation of Australia (101890). SA is supported by the Australian National Health and Medical Research Council (NHMRC) through an Overseas Early Career Fellowship (APP1139631).

## Supplementary files

Supplementary file 1. Search Strategy.

Supplementary file 2. RAMESES Publication Standards: Realist Syntheses.

Supplementary file 3. Studies Included in the Review, and Their Corresponding Right to Health Items.

Supplementary file 4. Formal Theoretical Perspectives.
