# Supplementary material for: Utility of the Right to Health for Addressing Skilled Health Worker Shortages in Low- and Middle-Income Countries
Source: Int J Health Policy Manag. 2022 Feb 7;11(11):2404–14. doi: 10.34172/ijhpm.2022.6168 (PMC9818093; doi:10.34172/ijhpm.2022.6168)
Supplement: Supplementary file 1 — Search Strategy. [file ijhpm-11-2404-s001.pdf]

Article title: Utility of the Right to Health for Addressing Skilled Health Worker Shortages in Low- and Middle-Income Countries

Journal name: International Journal of Health Policy and Management (IJHPM)

Authors' information: Kenneth Yakubu[1]*, Seye Abimbola[2], Andrea Durbach[3], Christine Balane[4], David Peiris[1], Rohina Joshi[5]

[1]The George Institute for Global Health, Faculty of Medicine, University of New South Wales, Sydney, NSW, Australia.

[2]School of Public Health, University of Sydney, Sydney, NSW, Australia.

[3]Australian Human Rights Institute, Faculty of Law, University of New South Wales, Sydney, NSW, Australia.

[4]Discipline of Paediatrics, School of Women's and Children's Health, University of New South Wales, Sydney, NSW, Australia.

[5]School of Population Health, Faculty of Medicine, University of New South Wales, Sydney, NSW, Australia

(*Corresponding author: kyakubu@georgeinstitute.org.au)

Supplementary file 1. Search Strategy

Search terms (Ovid – Medline, Embase, Global Health, Health Systems Evidence and PDQ-EVIDENCE)

1	exp "Emigration and Immigration"/
2	Health Personnel/ or Health Manpower/ or (((((((Health professional$ or health) adj2 provider$) or health) adj2 personnel) or health) adj2 practitioner$) or doctors or physicians or healthcare worker$ or health worker$ or nurse$ or nonphysician or non-physician or non physician or clinician$ or pharmac$ or midwi$).mp.
3	(recruit$ or retention or retain$ or regulat$).mp.
4	1 and 2 and 3
5	Developing Countries.sh,kf.
6	(Africa or Asia or Caribbean or West Indies or South America or Latin America or Central America).hw,kf,ti,ab,cp.
7	(Afghanistan or Albania or Algeria or Angola or Antigua or Barbuda or Argentina or Armenia or Armenian or Aruba or Azerbaijan or Bahrain or Bangladesh or Barbados or Benin or Byelarus or Byelorussian or Belarus or Belorussian or Belorussia or Belize or Bhutan or Bolivia or Bosnia or Herzegovina or Hercegovina or Botswana or Brasil or Brazil or Bulgaria or Burkina Faso or Burkina Fasso or Upper Volta or Burundi or Urundi or Cambodia or Khmer Republic or Kampuchea or Cameroon or Cameroons or Cameron or Camerons or Cape Verde or Central African Republic or Chad or Chile or China or Colombia or Comoros or Comoro Islands or Comores or Mayotte or Congo or Zaire or Costa Rica or Cote d'Ivoire or

	Ivory Coast or Croatia or Cuba or Cyprus or Czechoslovakia or Czech Republic or Slovakia or Slovak Republic or Djibouti or French Somaliland or Dominica or Dominican Republic or East Timor or East Timur or Timor Leste or Ecuador or Egypt or United Arab Republic or El Salvador or Eritrea or Estonia or Ethiopia or Fiji or Gabon or Gabonese Republic or Gambia or Gaza or Georgia Republic or Georgian Republic or Ghana or Gold Coast or Greece or Grenada or Guatemala or Guinea or Guam or Guiana or Guyana or Haiti or Honduras or Hungary or India or Maldives or Indonesia or Iran or Iraq or Isle of Man or Jamaica or Jordan or Kazakhstan or Kazakh or Kenya or Kiribati or Korea or Kosovo or Kyrgyzstan or Kirghizia or Kyrgyz Republic or Kirghiz or Kirgizstan or Lao PDR or Laos or Latvia or Lebanon or Lesotho or Basutoland or Liberia or Libya or Lithuania or Macedonia or Madagascar or Malagasy Republic or Malaysia or Malaya or Malay or Sabah or Sarawak or Malawi or Nyasaland or Mali or Malta or Marshall Islands or Mauritania or Mauritius or Agalega Islands or Mexico or Micronesia or Middle East or Moldova or Moldovia or Moldovian or Mongolia or Montenegro or Morocco or Ifni or Mozambique or Myanmar or Myanma or Burma or Namibia or Nepal or Netherlands Antilles or New Caledonia or Nicaragua or Niger or Nigeria or Northern Mariana Islands or Oman or Muscat or Pakistan or Palau or Palestine or Panama or Paraguay or Peru or Philippines or Philipines or Phillipines or Phillippines or Poland or Portugal or Puerto Rico or Romania or Rumania or Roumania or Russia or Russian or Rwanda or Ruanda or Saint Kitts or St Kitts or Nevis or Saint Lucia or St Lucia or Saint Vincent or St Vincent or Grenadines or Samoa or Samoan Islands or Navigator Island or Navigator Islands or Sao Tome or Saudi Arabia or Senegal or Serbia or Montenegro or Seychelles or Sierra Leone or Slovenia or Sri Lanka or Ceylon or Solomon Islands or Somalia or South Africa or Sudan or Suriname or Surinam or Swaziland or Syria or Tajikistan or Tadzhikistan or Tadjikistan or Tadzhik or Tanzania or Thailand or Togo or Togolese Republic or Tonga or Trinidad or Tobago or Tunisia or Turkey or Turkmenistan or Turkmen or Uganda or Ukraine or Uruguay or USSR or Soviet Union or Union of Soviet Socialist Republics or Uzbekistan or Uzbek or Vanuatu or New Hebrides or Venezuela or Vietnam or Viet Nam or West Bank or Yemen or Yugoslavia or Zambia or Zimbabwe or Rhodesia).hw,kf,ti,ab,cp.
8	((developing or less* developed or under developed or underdeveloped or middle income or low* income or underserved or under served or deprived or poor*) adj (countr* or nation? or population? or world)).ti,ab.
9	((developing or less* developed or under developed or underdeveloped or middle income or low* income) adj (economy or economies)).ti,ab.
10	(low* adj (gdp or gnp or gross domestic or gross national)).ti,ab.
11	(low adj3 middle adj3 countr*).ti,ab.
12	(lmic or lmics or third world or lami countr*).ti,ab.
13	transitional countr*.ti,ab.
14	or/5-13

15	4 AND 14

**ProQuest**
"Emigration and Immigration"/ AND Health Personnel/ or Health Manpower/ or (((((((Health professional$ or health) adj2 provider$) or health) adj2 personnel) or health) adj2 practitioner$) or doctors or physicians or healthcare worker$ or health worker$ or nurse$ or nonphysician or non-physician or non physician or clinician$ or pharmac$ or midwi$).mp. AND (recruit$ or retention or retain$ or regulat$).mp.

**Scopus**
(TITLE-ABS-KEY ((emigration OR immigration)) AND ALL ((health AND personnel OR health AND manpower OR health AND professional OR health AND provider OR health AND personnel OR health AND practitioner OR doctors OR physicians OR healthcare AND worker OR health AND worker OR nurse OR non-physician OR non AND physician OR clinicial OR pharmacy)) AND ALL ((recruit OR retention OR retain OR regular)) AND ALL ((africa OR asia OR caribbean OR west AND indies OR south AND america OR latin AND america OR central AND america)))

**Web of science**
(emigration or immigration) AND ALL FIELDS: (Health Personnel OR Health Manpower OR Health professional OR health provider OR health personnel OR health practitioner OR doctors OR physicians OR healthcare worker OR health worker OR nurse OR nonphysician OR non-physician OR non physician OR clinician OR pharmacy OR midwi) AND ALL FIELDS: ((recruit OR retention OR retain OR regular)) AND ALL FIELDS:((Africa or Asia or Caribbean or West Indies or South America or Latin America or Central America))
Timespan: All years. Indexes: SCI-EXPANDED, SSCI, A&HCI, CPCI-S, CPCI-SSH, BKCI-S, BKCI-SSH, ESCI, CCR-EXPANDED, IC.

**CINAHL**
(emigration or immigration or migration) AND Health Personnel/ OR Health Manpower/ OR Health professional OR health provider OR health personnel OR health practitioner OR doctors OR physicians OR healthcare worker OR health worker OR nurse OR nonphysician OR non-physician OR non physician OR clinician OR pharmacy OR midwi AND (recruit$ or retention or retain$ or regulat) AND (Africa or Asia or Caribbean or West Indies or South America or Latin America or Central America)

**APAIS-Health**
(ALLTERMS,FC:emigration ALLTERMS,FC:or ALLTERMS,FC:immigration ALLTERMS,FC:or ALLTERMS,FC:migration) AND (ALLTERMS,FC:health ALLTERMS,FC:care ALLTERMS,FC:professionals OR ALLTERMS,FC:health ALLTERMS,FC:care ALLTERMS,FC:providers OR ALLTERMS,FC:health ALLTERMS,FC:care ALLTERMS,FC:personnel OR ALLTERMS,FC:health ALLTERMS,FC:care ALLTERMS,FC:practitioners OR ALLTERMS,FC:doctors OR ALLTERMS,FC:physicians OR ALLTERMS,FC:healthcare ALLTERMS,FC:workers OR

ALLTERMS,FC:health ALLTERMS,FC:workers OR ALLTERMS,FC:nurses OR ALLTERMS,FC:non ALLTERMS,FC:physician ALLTERMS,FC:clinicians OR ALLTERMS,FC:clinicians OR ALLTERMS,FC:pharmacists OR ALLTERMS,FC:midwives) AND (ALLTERMS,FC:recruit OR ALLTERMS,FC:retention OR ALLTERMS,FC:regulation)

**First Google Scholar Search**
(Emigration OR Immigration) AND (Health professionals or health care providers or health care personnel or health care practitioners or doctors or physicians or healthcare workers or health workers or nurses or non physician clinicians or clinicians or pharmacists or midwi*) AND recruit$ AND (retention or retaining) AND (developing or less* developed or under developed or underdeveloped or middle income or low* income or underserved or underserved or deprived or poor* OR Afghanistan or Albania or Algeria or Angola or Antigua or Barbuda or Argentina or Armenia or Armenian or Aruba or Azerbaijan or Bahrain or Bangladesh or Barbados or Benin or Byelarus or Byelorussian or Belarus or Belorussian or Belorussia or Belize or Bhutan or Bolivia or Bosnia or Herzegovina or Hercegovina or Botswana or Brasil or Brazil or Bulgaria or Burkina Faso or Burkina Fasso or Upper Volta or Burundi or Urundi or Cambodia or Khmer Republic or Kampuchea or Cameroon or Cameroons or Cameron or Camerons or Cape Verde or Central African Republic or Chad or Chile or China or Colombia or Comoros or Comoro Islands or Comores or Mayotte or Congo or Zaire or Costa Rica or Cote d'Ivoire or Ivory Coast or Croatia or Cuba or Cyprus or Czechoslovakia or Czech Republic or Slovakia or Slovak Republic or Djibouti or French Somaliland or Dominica or Dominican Republic or East Timor or East Timur or Timor Leste or Ecuador or Egypt or United Arab Republic or El Salvador or Eritrea or Estonia or Ethiopia or Fiji or Gabon or Gabonese Republic or Gambia or Gaza or Georgia Republic or Georgian Republic or Ghana or Gold Coast or Greece or Grenada or Guatemala or Guinea or Guam or Guiana or Guyana or Haiti or Honduras or Hungary or India or Maldives or Indonesia or Iran or Iraq or Isle of Man or Jamaica or Jordan or Kazakhstan or Kazakh or Kenya or Kiribati or Korea or Kosovo or Kyrgyzstan or Kirghizia or Kyrgyz Republic or Kirghiz or Kirgizstan or Lao PDR or Laos or Latvia or Lebanon or Lesotho or Basutoland or Liberia or Libya or Lithuania or Macedonia or Madagascar)

**Second Google Scholar Search**
(Emigration OR Immigration) AND (Health professionals or health care providers or health care personnel or health care practitioners or doctors or physicians or healthcare workers or health workers or nurses or non physician clinicians or clinicians or pharmacists or midwi*) AND recruit$ AND (retention or retaining) AND (developing or less* developed or under developed or underdeveloped or middle income or low* income or underserved or underserved or deprived or poor* or Malagasy Republic or Malaysia or Malaya or Malay or Sabah or Sarawak or Malawi or Nyasaland or Mali or Malta or Marshall Islands or Mauritania or Mauritius or Agalega Islands or Mexico or Micronesia or Middle East or Moldova or Moldovia or Moldovian or Mongolia or Montenegro or Morocco or Ifni or Mozambique or Myanmar or Myanma or Burma or Namibia or Nepal or Netherlands Antilles or New Caledonia or Nicaragua or Niger or Nigeria or Northern Mariana Islands or Oman or Muscat or Pakistan or Palau or Palestine or Panama or Paraguay or Peru or Philippines or Philipines or Phillipines or Phillippines or Poland or Portugal or Puerto Rico or Romania or Rumania or Roumania or Russia or Russian or

Rwanda or Ruanda or Saint Kitts or St Kitts or Nevis or Saint Lucia or St Lucia or Saint Vincent or St Vincent or Grenadines or Samoa or Samoan Islands or Navigator Island or Navigator Islands or Sao Tome or Saudi Arabia or Senegal or Serbia or Montenegro or Seychelles or Sierra Leone or Slovenia or Sri Lanka or Ceylon or Solomon Islands or Somalia or South Africa or Sudan or Suriname or Surinam or Swaziland or Syria or Tajikistan or Tadzhikistan or Tadjikistan or Tadzhik or Tanzania or Thailand or Togo or Togolese Republic or Tonga or Trinidad or Tobago or Tunisia or Turkey or Turkmenistan or Turkmen or Uganda or Ukraine or Uruguay or USSR or Soviet Union or Union of Soviet Socialist Republics or Uzbekistan or Uzbek or Vanuatu or New Hebrides or Venezuela or Vietnam or Viet Nam or West Bank or Yemen or Yugoslavia or Zambia or Zimbabwe or Rhodesia)

**WHO**
tw:((tw:((recruit* OR retention OR retain*))) AND (tw:(migration)) AND (tw:((health personnel OR health manpower OR (health professionals OR health care providers OR health care personnel OR health care practitioners OR doctors OR physicians OR healthcare workers OR health workers OR nurses OR non physician clinicians OR clinicians OR pharmacists OR midwi*))) AND (tw:((developing OR less* developed OR under developed OR underdeveloped OR middle income OR low* income)) OR (africa OR asia OR caribbean OR west indies OR south america OR latin america OR central america) OR (lmic OR lmics OR third world OR lami countr*)))) AND (instance:"ghl")
