# Supplementary material for: Utility of the Right to Health for Addressing Skilled Health Worker Shortages in Low- and Middle-Income Countries
Source: Int J Health Policy Manag. 2022 Feb 7;11(11):2404–14. doi: 10.34172/ijhpm.2022.6168 (PMC9818093; doi:10.34172/ijhpm.2022.6168)
Supplement: Supplementary file 2 — RAMESES Publication Standards: Realist Syntheses. [file ijhpm-11-2404-s002.pdf]

**Article title:** Utility of the Right to Health for Addressing Skilled Health Worker Shortages in Low- and Middle-Income Countries

**Journal name:** International Journal of Health Policy and Management (IJHPM)

**Authors' information:** Kenneth Yakubu[1]*, Seye Abimbola[2], Andrea Durbach[3], Christine Balane[4], David Peiris[1], Rohina Joshi[5]

[1]The George Institute for Global Health, Faculty of Medicine, University of New South Wales, Sydney, NSW, Australia.

[2]School of Public Health, University of Sydney, Sydney, NSW, Australia.

[3]Australian Human Rights Institute, Faculty of Law, University of New South Wales, Sydney, NSW, Australia.

[4]Discipline of Paediatrics, School of Women's and Children's Health, University of New South Wales, Sydney, NSW, Australia.

[5]School of Population Health, Faculty of Medicine, University of New South Wales, Sydney, NSW, Australia

(*Corresponding author: kyakubu@georgeinstitute.org.au)

**Supplementary file 2.** RAMESES Publication Standards: Realist Syntheses.

**4. Main content:**

| **Supplementary file 2.** RAMESES Publication Standards: Realist Syntheses. | |
|---|---|
| **1. Title**: In the title, identify the document as a realist synthesis or review | This has been provided on page 1, line 2 |
| **2. Abstract:** While acknowledging publication requirements and house style, abstracts should ideally contain brief details of: the study's background, review question or objectives; search strategy; methods of selection, appraisal, analysis, and synthesis of sources; main results; and implications for practice. | We have followed the journals requirement for writing the abstract which also addressed the RAMESES requirements. See pages 1 and 2 |
| **3. Introduction**: Rationale for review. Explain why the review is needed and what it is likely to contribute to existing understanding of the topic area. | This has been described starting from Line 3 on page 3, to Line 7 on page 5 |
| **4. Objectives and focus of review**: State the objective(s) of the review and/or the review question(s). Define and provide a rationale for the focus of the review. | This has been stated, starting from Line 4 to Line 5 on page 5. |
| **5. Methods:** Changes in the review process Any changes made to the review process that was initially planned should be briefly described and justified. | The review process has remained the same as registered on PROSPERO. (CRD42019139372). |
| **6. Rationale for using realist synthesis:** Explain why realist synthesis was considered the most appropriate method to use. | Starting from Line 24 on page 4, to line 4/5 on page 5; we provided the rationale for using the realist approach. |
| **7. Scoping the literature**: Describe and justify the initial process of exploratory scoping of the literature. | Starting from Page 5, Line 15, to Line 21; we described the process for designing the initial program theory. |
| **8. Searching processes:** While considering specific requirements of the journal or other publication outlet, state and provide a rationale for how the iterative searching was done. Provide details on all the sources accessed for information in the review. Where searching in electronic databases has taken place, the details should include, for example, name of database, search terms, dates of coverage, and date last searched. If individuals familiar with the relevant literature and/or topic area were contacted, indicate how they were identified and selected. | Starting from Line 6 to Line 15 on Page 6, we described the search process and sources of data for designing the final program theory. |

| | |
|---|---|
| **9. Selection and appraisal of documents** Explain how judgements were made about including and excluding data from documents and justify these. | Starting from Line 2 to Line 8 on page 7,<br>we described the inclusion and exclusion criteria. We also described the quality appraisal process we undertook on line 10-17 on page 7. |
| **10. Data extraction:** Describe and explain which data or information were extracted from the included documents and justify this selection. | Starting from Line 20 on page 8, to Line 9 on page 9, we described the data extraction process. |

| | |
|---|---|
| **11. Analysis and synthesis** Describe the analysis and synthesis in detail. This section should information on the constructs analysed and describe the analytic process. | Starting from Line 19 on page 7 to line 12 on page 8, we described how we analyzed the data and synthesized the evidence. |
| **12. Results document flow diagram:** Provide details on the number of documents assessed for eligibility and included in the review with reasons for exclusion at each stage as well as an indication of their source of origin (e.g. from searching databases, templates (which are likely to modification to suit the data) that are provided. | A document flow diagram has been provided on page 11, line 6-16 |
| **13. Document characteristics** Provide information on the characteristics of the documents included in the review. | This has been provided in Appendix 2 in the supplementary file |
| **14. Main findings**: Present the key with a specific focus on theory building testing. | We have presented the results with a focus on theory building starting table 4 on page 14 (the CMO configuration), and the main text between pages 14 to page 21 |
| **15. Discussion:** Summary of Summarize the main findings, taking into account the review's objective(s), research question(s), focus, and intended audience(s). | A summary of the findings has been provided starting from line 26 to 30 on page 18 and lines 1-7 on page 19. |

*Publication Guidelines by Wong et al. BMC Medicine 2013, 11:21*
