# Supplementary material for: Utility of the Right to Health for Addressing Skilled Health Worker Shortages in Low- and Middle-Income Countries
Source: Int J Health Policy Manag. 2022 Feb 7;11(11):2404–14. doi: 10.34172/ijhpm.2022.6168 (PMC9818093; doi:10.34172/ijhpm.2022.6168)
Supplement: Supplementary file 3 — Studies Included in the Review, and Their Corresponding Right to Health Items. [file ijhpm-11-2404-s003.pdf]

Article title: Utility of the Right to Health for Addressing Skilled Health Worker Shortages in Low- and Middle-Income Countries

Journal name: International Journal of Health Policy and Management (IJHPM)

Authors' information: Kenneth Yakubu[1]*, Seye Abimbola[2], Andrea Durbach[3], Christine Balane[4], David Peiris[1], Rohina Joshi[5]

[1]The George Institute for Global Health, Faculty of Medicine, University of New South Wales, Sydney, NSW, Australia.

[2]School of Public Health, University of Sydney, Sydney, NSW, Australia.

[3]Australian Human Rights Institute, Faculty of Law, University of New South Wales, Sydney, NSW, Australia.

[4]Discipline of Paediatrics, School of Women's and Children's Health, University of New South Wales, Sydney, NSW, Australia.

[5]School of Population Health, Faculty of Medicine, University of New South Wales, Sydney, NSW, Australia

(*Corresponding author: kyakubu@georgeinstitute.org.au)

Supplementary file 3. Studies Included in the Review, and Their Corresponding Right to Health Items

4. Main content:

Citation	Study type	Countries involved & income status	Right to Health (RTH) items
Abuagla A., Badr E. (2016)[26]	Mixed methods (key informant interviews + Document Reviews)	Sudan (LMIC)<br>Libya (UMIC)<br>Saudi Arabia (HIC)	RTH 8, 9, 12
Dovlo D. (2003)[27]	Case study	Ethiopia (LICs)<br>Ghana (LMIC)<br>Botswana (UMIC)<br>Cuba (UMIC)<br>Jamaica (UMIC)	RTH 8, 9, 11, 12
Sato C. (2012) [28]<br><br>Connell J., Stilwell B. (2006) [29]	Working Paper Series<br><br>Book Section	South Africa (UMIC)<br>The Caribbean (MICs)<br>Britain (HIC)	RTH 8, 11, 12
Edge J.S., Hoffman S.J. (2013)[30]	Survey of key informants	Australia (HIC)<br>Canada (HIC)<br>United Kingdom (HIC)<br>United States of America (HIC)	RTH 12

Plotnikova E.V. (2012)[31]	Qualitative Content Analysis of Policy Documents	LMICs in general<br>Philippines (LMIC)<br>India (LMIC)<br>South Africa (UMIC)<br>United Kingdom (HIC)	RTH 8, 11, 12
Bevan S. (2005)[32]	Report	United Kingdom (HIC)<br>South Africa (LMIC)	RTH 12
Willets A., Martineau T. (2006)[33]	Report	United Kingdom (HIC)<br>Sub-Saharan Africa (LMICs)<br>South Africa (UMIC)	RTH 12
Dauphinee WD. (2005) [34]	Commentary	Canada (HIC)<br>South Africa (UMICs)	RTH 12
Adhikari R., Grigulis A. (2014) [35]	Qualitative study (in-depth interviews)	Malawi (LIC)<br>Nepal (LIC)<br>United Kingdom (HIC)	RTH 12
Blacklock et al (2012) [36]	Time series analysis	India (LMIC)<br>Philippines (LMIC)<br>South Africa (UMIC)<br>United Kingdom (HIC)	RTH 11, 12
Buchan, J. (2002)[37]	Case study	United Kingdom (HIC)<br>South Africa (UMIC)<br>The Caribbean (MICs)	RTH 12
Buchan J. et al (2009) [38]	Case studies	Kenya (LMIC)<br><br>LMICs in general<br>South Africa (UMIC)<br>United Kingdom (HIC)<br><br>Ghana (LMIC)	RTH 5, 8, 9,12
Squires A. et al (2016) [39]	Random effects panel data analysis	United States (HICs)	RTH 8
Tankwanchi et al (2019)[40]	Secondary analysis of health worker migration trends	Western Countries (HICs)<br><br>South Africa (UMIC)<br>Southern Africa countries (LMICs)<br>OECD countries (HICs)<br><br>United Kingdom (HIC)	RTH 8, 9,11, 12
Herfs PG. (2014) [41]	Qualitative study (structured questionnaires)	United Kingdom (HIC)<br>Netherlands (HIC)<br>Tanzania (LIC)<br>Kenya (LMIC)<br>Nigeria (LMIC)<br>South Africa (UMIC), Afghanistan (LIC),<br>Iraq (UMIC),<br>Romania (UMIC) and<br>Surinam (UMIC)	RTH 12
Tankwanchi AB. et al (2015)[42]	Uncontrolled Before-and-After study	United States of America (HIC)<br>Sub-Saharan Africa (LMICs)	RTH 8, 12
Peñaloza B. et al (2011)[43]	Systematic review	USA (HIC)<br>LMICs	N/A
Kober K. et al (2004)[44]	Case study	Mozambique (LIC)<br>Malawi (LIC)<br>Cuba (UMIC)<br>South Africa (UMIC)<br>Southern African Development Community (SADC)<br>International NGO (Médecins Sans Frontières)	RTH 8,9, 11,12

Moullan Y. (2009) [46]	Analysis paper	LMICs in general<br>Organisation for Economic Cooperation and Development (OECD) countries. (HICs)	RTH 4, 8, 9, 11
Schulze et al. (2014)[47]	Editorial	Malawi (LIC)<br>Germany (HIC)	RTH 8, 11,12
Ossai EN. et al (2012) [48]	Systematic review	Mali (LIC),<br>Santé Sud (International NGO)	RTH 8, 9
Sherr K. et al (2012)[49]	Observational prospective study	Mozambique (LIC)<br>International NGOs	RTH 5, 8, 11
Barnighausen T. et al (2009)[50]	Cost Benefit Analysis	Sub-Saharan Africa	RTH 8, 9
Cailhol J. et al (2013) [51]	Mixed methods (semi-structured interviews + analysis of secondary data)	Angola (LMIC)<br>Burundi (LIC)<br>Lesotho (LIC)<br>Mozambique (UMIC)<br>South Africa (UMIC)<br>United States (HIC)<br>International NGOs	RTH 5, 8, 9 11
Shaffer FA. et al (2016)[52]	Case study	United States (HICs)<br>Philippines (LMIC)<br>India (LMIC)	RTH 12
Mandeville KL. et al (2015, 2016)[53,54]	Retrospective Cohort Study<br><br>Discrete Choice Experiment	Malawi (LIC)<br>Britain (HIC)<br>South Africa (UMIC)<br>Germany (HIC)	RTH 6, 8, 9, 11,12
Brush BL. et al (2007)[55]	Analysis paper	Philippines (LMIC)<br>United Kingdom (HIC)	RTH 12
Yan J. et al (2006) [56]<br><br>Salmon ME. et al (2007)[65]	Conference presentation<br><br>Contextual Analysis	International NGO<br>Caribbean region (MICs)<br>St Vincent (UMIC)<br>United States of America (HIC)<br>Antigua (HIC)<br>Grenada (UMIC)<br>Jamaica (UMICs)<br>United States (HIC)<br>Canada (HIC)	RTH 8, 11,12
Geber H. (2013) [57]	Program report	South Africa (UMIC)<br>International NGO - CARTA (The Consortium for Advanced Research Training in Africa is a project within the APHRC. The project, funded by Wellcome Trust).	RTH 8, 11
Zijlstra EE. et al (2007)[58]	Analysis paper	Norway, Netherlands, Sweden and the UK (HICs)<br>Malawi (LIC)	RTH 8, 11
Dambisya Y. et al (2007)[59]	Review	Botswana (UMIC)<br>Lesotho (LIC)<br>Mozambique (LIC)<br>Malawi (LIC)<br>Tanzania (LIC)<br>Uganda<br>Zambia (LMIC)	RTH 8, 9, 11,12

		Eswatini (LIC)<br>Mauritius (UMIC)<br>*East and Southern Africa (LMICs)<br>National, Regional and International NGOs (CHAM, Southern Africa Capacity initiative, WHO, UNDP)	
Henderson LN. et al (2008)[60]	Review	Papua New Guinea (LMIC)<br>Vietnam (LMIC)<br>Thailand (UMIC)<br>Cambodia (LMIC)<br>Philippines (LMIC)<br>International NGO (Global fund)	RTH 8, 9,11,12
Kober K., Van Damme W. (2006)[61]	Case study	Eswatini (LIC)<br>International NGOs (WHO, Global Fund)	RTH 8, 9, 11
Tambulasi R.I.C. (2012)[63]	Analysis paper	Malawi (LIC)<br>International NGOs	RTH 8, 9, 11
Reid S.J. (2001)[60]	Mixed methods (Qualitative semi-structured interviews + cross sectional surveys)	South Africa (UMIC)	RTH 5,8,12
Chimbari M. et al (2008)[66]	Cross-sectional survey	Zimbabwe (LMIC)	RTH 8,9,11,12
Mufunda J. et al (2007)[67]	Program evaluation	Zimbabwe (LMIC)	RTH 5, 8, 9
Iipinge S. et al (2009)[68]	Case studies	Lesotho (LIC)<br>Malawi (LIC)<br>Tanzania (LIC)<br>Malawi (LIC)<br>Eswatini (LIC)<br>Mozambique (LIC)<br>Zambia (LMIC)<br>Mauritius (UMIC)<br>East and Southern African region (LMICs)	RTH 8, 9, 11,12
Masango S. et al (2008)[69]	Case study	Eswatini (LIC)	RTH 8,9
van Rensburg, H.C.J. (2014)[70]	Analysis paper	South Africa (UMIC)	RTH 1, 5, 8, 11,12
Darko VM. et al (2006)[71]	Prospective study	Ghana (LMIC)	RTH 8, 12
George A. et al (2019)[72]	Cross-sectional survey	South Africa (UMIC)	RTH 1, 5, 8, 12
Paina L. et al (2016)[73]	Analysis paper	Romania (LMIC)	RTH 5,8,9,12
Hammett D. (2014)[74]	Qualitative study (individual interviews)	South Africa (UMIC)<br>Cuba<br>(UMIC)	RTH 5,8,9,11,12
Kanchanachitra C. et al (2011)[75]	Review	Cambodia (LMIC)<br>Laos (LMIC)<br>Myanmar (LMIC)<br>Thailand (UMIC)<br>Indonesia (LMIC)<br>Philippines (LMIC)	RTH 1,5, 8,9,10,12
Walton-Roberts M. et al (2017)[76]	Mixed methods (scoping review, survey and interviews)	India (LMIC)	RTH 12
George G. et al (2012)[77]	Review	South Africa (UMIC)	RTH 8

Chirwa Y. et al (2014)[78]	Review	Zimbabwe (LMIC)	RTH 5, 8, 9, 12
Mullan F. et al (2011)[79]	Cross sectional survey	Mali (LIC)<br>France (HIC)<br>Sub-Saharan Africa (LMICs)<br>Europe (HICs)<br>North America (HIC)<br>Mozambique (LIC)<br>South Africa (UMIC)<br>Ethiopia (LIC)<br>Nigeria (LMIC)	RTH 8,9,11,12
van de Pas R et al (2016)[80]	Case studies.	East Africa (LMICs)<br>Southern Africa (LMICs)<br>Europe (HIC)	RTH 12
Manning C. et al (2007)[81]	Reports	Thailand (UMIC)<br>Indonesia (LMIC)<br>Philippines (LMIC)<br>Cambodia (LMIC)	RTH 8, 12
Efendi F., Chen C. (2014)[82]	Poster presentation	Indonesia (LMIC)	RTH 12
Lassey AT. et al (2013)[83]	Cross-sectional survey	Ghana (LMIC)	RTH 8,9
Wiwanitkit V. (2011)[84]	Policy report	Thailand (UMIC)	RTH 5, 6, 8, 9, 12
Enlinson M., Mazetto D. (2018)[85]<br><br>Harris M. (2016)[86]	Program evaluation	Brazil (UMIC)<br>Cuba (UMIC)	RTH 1, 5 8,9,11
Bode CO. et al (2013)[87]	Cross-sectional survey	West Africa (LMICs)	RTH 8, 9,11
Derbew M. et al (2016) [88]	Cross sectional survey	Ethiopia (LIC)	RTH 8
Labonte R. et al. (2015) [89]	Mixed methods (scoping review, key informant interviews)	South Africa (UMIC)<br>Cuba (UMIC)<br>United States of America (HIC)<br>Southern Africa Development Community (LMICs)	RTH 5, 8, 9,11,12
Hongoro C., McPake B. (2004)[90]	Report	Philippines (LMIC)<br>Ghana (LMIC)<br>South Africa (UMIC)<br>Thailand (UMIC)<br>Australia (HIC)<br>UK (HIC)	RTH 8, 9,11, 12
Willis-Shattuck M. et al (2008) [92]	Systematic review	Bangladesh (LMIC)<br>Kazakhstan (UMIC)<br>South Africa (UMIC)<br>Cameroon (LMIC)<br>Zimbabwe (LMIC)<br>Uganda (LIC)<br>Mali (LIC)	RTH 8,9
Ndetei DM. et al (2008) [92]	Mixed methods (survey, key informant interviews, analysis of secondary data)	Kenya (LMIC)	RTH 8, 9, 11,12
Oman KM. et al (2009)[93]	Qualitative study (semi-structured interviews)	Fiji (UMIC)<br>Countries in the Pacific Island (LMICs)	RTH 8, 12

Key: LIC – low income country, LMIC- low- and middle-income country, and HIC – high income country.
