# Supplementary material for: Utility of the Right to Health for Addressing Skilled Health Worker Shortages in Low- and Middle-Income Countries
Source: Int J Health Policy Manag. 2022 Feb 7;11(11):2404–14. doi: 10.34172/ijhpm.2022.6168 (PMC9818093; doi:10.34172/ijhpm.2022.6168)
Supplement: Supplementary file 4 — Formal Theoretical Perspectives. [file ijhpm-11-2404-s004.pdf]

Article title: Utility of the Right to Health for Addressing Skilled Health Worker Shortages in Low- and Middle-Income Countries

Journal name: International Journal of Health Policy and Management (IJHPM)

Authors' information: Kenneth Yakubu[1]*, Seye Abimbola[2], Andrea Durbach[3], Christine Balane[4], David Peiris[1], Rohina Joshi[5]

[1]The George Institute for Global Health, Faculty of Medicine, University of New South Wales, Sydney, NSW, Australia.

[2]School of Public Health, University of Sydney, Sydney, NSW, Australia.

[3]Australian Human Rights Institute, Faculty of Law, University of New South Wales, Sydney, NSW, Australia.

[4]Discipline of Paediatrics, School of Women's and Children's Health, University of New South Wales, Sydney, NSW, Australia.

[5]School of Population Health, Faculty of Medicine, University of New South Wales, Sydney, NSW, Australia

(*Corresponding author: kyakubu@georgeinstitute.org.au)

Supplementary file 4. Formal Theoretical Perspectives

4. Main content (description method):

For the abduction stage of the realist analysis (see Box 1), we conducted a search in Google Scholar and considered the first 300 results. See the paper by Haddaway NR (2015) for more information on why we considered this sufficient.[1] There were 53 articles that we considered useful for helping us explain our results. Eventually, we found three sets of theoretical perspectives which we considered relevant for interpreting the emerging context-outcome pairs:
1. **Collective action:** on this, Jagers et al refers to Olson's definition of collective action problems as a scenario wherein the short term interest of stakeholders is in conflict with long-term interest of the collective, thus hindering realisation of a collective

benefit.[2] Jagers observed that state actors will choose collective action if their reputation is at stake. This choice is further enabled if and where there is a social norm that encourages (and “punishes” deviation from) the desired behaviour. When there is a high level of initial cooperation, more state actors will choose reciprocity, which can enable trust and reinforce a cycle of collective action.
2. **Norm conformity or violation:** this arose primarily from Shannon’s description of why state actors conform with, or violate norms.[3] Established norms set collective expectations for proper behaviour among actors with a given identity. Hence, state actors will seek to conform with these expectations because they offer a useful heuristic for guiding interaction, or they will seek to comply just to restore and maintain social approval, or yet seek to comply because it aligns with their set of values and need for self-esteem. If a conflict arises between the perceived national interest of state actors and a given international norm, and the state actors value their international reputation, then they will seek to justify violation of a specific norm if there are gaps in its description of a desired behaviour, or of it fails to specify what is acceptable in different situations.
3. **Transnational Legal Process:** this originated from Koh’s description of why state actors comply with global human rights norms. Koh theorized that social interactions which occur at international fora raise awareness about human rights norms. It also provides the opportunity to recruit more state actors into a process of accepting human rights norms as relevant and applicable to a given situation.[4] Through repeated interactions, an interpretation that is relevant to the situation emerges among state actors. This theory emphasises the translation of global human rights norms (at the top) into the domestic structure of individual countries (the bottom), and is triggered

by transnational actors who evoke interactions.[4] However, legal, political or social integration of global human rights norms into the domestic structure of countries is not a common culmination of a global top-bottom mechanism.[5]

References

1. Haddaway NR, Collins AM, Coughlin D, Kirk S. The Role of Google Scholar in Evidence Reviews and Its Applicability to Grey Literature Searching. *PLOS ONE.* 2015;10(9):e0138237. doi:10.1371/journal.pone.0138237

2. Jagers SC, Harring N, Lofgren A, et al. On the preconditions for large-scale collective action. *Ambio.* 2020;49(7):1282-1296. doi:10.1007/s13280-019-01284-w

3. Shannon VP. Norms Are What States Make of Them: The Political Psychology of Norm Violation. *International Studies Quarterly.* 2000;44(2):293-316.

4. Koh HH. Why Do Nations Obey International Law? *The Yale Law Journal.*106:2599-2659.

5. Hammonds R, Ooms G, Vandenhole W. Under the (legal) radar screen: global health initiatives and international human rights obligations. *BMC International Health and Human Rights.* 2012;12.
